# Disruption of Neutrophil Extracellular Traps (NETs) Links Mechanical Strain to Post-traumatic Inflammation

**DOI:** 10.3389/fimmu.2019.02148

**Published:** 2019-10-24

**Authors:** Shailesh Agarwal, Shawn J. Loder, David Cholok, John Li, Guowu Bian, Srilakshmi Yalavarthi, Shuli Li, William F. Carson, Charles Hwang, Simone Marini, Chase Pagani, Nicole Edwards, Matthew J. Delano, Theodore J. Standiford, Jason S. Knight, Steven L. Kunkel, Yuji Mishina, Peter A. Ward, Benjamin Levi

**Affiliations:** ^1^Department of Surgery, University of Michigan Medical School, Ann Arbor, MI, United States; ^2^Department of Medicine, University of Michigan Medical School, Ann Arbor, MI, United States; ^3^Department of Pathology, University of Michigan Medical School, Ann Arbor, MI, United States; ^4^Department of Biologic and Materials Sciences & Prosthodontics, University of Michigan School of Dentistry, Ann Arbor, MI, United States

**Keywords:** neutrophils, NET, trauma, movement, inflammation

## Abstract

Inflammation after trauma is both critical to normal wound healing and may be highly detrimental when prolonged or unchecked with the potential to impair physiologic healing and promote *de novo* pathology. Mechanical strain after trauma is associated with impaired wound healing and increased inflammation. The exact mechanisms behind this are not fully elucidated. Neutrophil extracellular traps (NETs), a component of the neutrophil response to trauma, are implicated in a range of pro-inflammatory conditions. In the current study, we evaluated their role in linking movement and inflammation. We found that a link exists between the disruption and amplification of NETs which harbors the potential to regulate the wound's response to mechanical strain, while leaving the initial inflammatory signal necessary for physiologic wound healing intact.

## Introduction

Musculoskeletal trauma poses a unique challenge due to the need for early mobilization to facilitate rehabilitation. However, movement also up-regulates local inflammation which portends a poor wound healing prognosis (1–6). Therefore, immobilization or “resting” of an injured joint is often implemented to reduce local inflammation and edema (7–9). The mechanism for this local effect of immobilization remains poorly characterized. Although anti-inflammatory therapeutics such as corticosteroids or non-steroidal anti-inflammatory drugs (NSAIDs) reduce systemic inflammation (10), they do not target pathways specific to the pro-inflammatory effects of mechanical strain; additionally, these drugs cause well-recognized adverse effects including poor wound healing, diabetes, hyperlipidemia, and gastrointestinal toxicity (10–12). Identification of specific pathways through which mechanical strain propagates the inflammatory response would expand therapeutic strategies to mitigate acute inflammation, prevent pathologic wound healing, reduce time to rehabilitation, and improve patient recovery. In addition to musculoskeletal trauma, these findings may influence treatment for a broad spectrum of pathologies mediated by mechanical strain (13–15).

Neutrophil extracellular traps (NETs), discrete structural “webs” composed of DNA and citrullinated histones released by neutrophils, have been studied for their role in both septic and aseptic inflammation (16–26). Several models of infection have demonstrated that NETs provide a protective effect, likely through bacterial trapping (27). Other studies have suggested that NETs are causative factors for inflammatory conditions including lupus (16–19), rheumatoid arthritis (20, 21), non-healing diabetic wounds (28), and vascular thromboses (22). However, the role of NETs as a link between mechanical strain and exacerbated inflammation remains unclear. Because NETs are composed of macromolecules with expansive structural features, we hypothesize that movement mediates inflammation through physical disruption of these structures and subsequent expansion of the NET superstructure. This leads to a phenomenon we describe as “NET-induced NETosis,” a type of secondary NET formation by which mechanical stress is capable of independently propagating the inflammatory process following the inciting inflammatory event.

## Results

**Figure 1:**
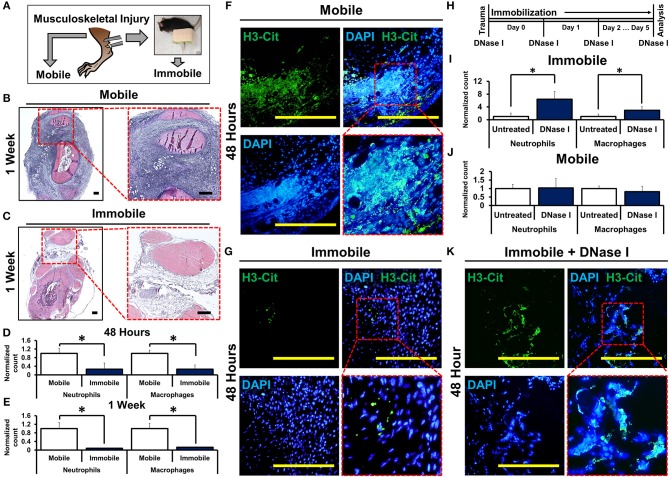
Mechanical or chemical disruption of NETs augments inflammation and induces pathologic wound healing. **(A)** Mice undergo tendon transection and either remain mobile or are immobilized via splint; **(B)** H&E staining of mobile hindlimb 1 week post-injury intense blue staining represent infiltrated cells casting (4x *left*; 10x *right*); **(C)** H&E staining of immobile hindlimb 1 week post-injury; **(D)** Immobile hindlimbs (*n* = 6) have significantly reduced normalized neutrophil (1.0 vs. 0.27, *p* < 0.05) and macrophage (1.0 vs. 0.26, *p* < 0.05) counts compared with mobile hindlimbs (*n* = 6) 48 h after injury; **(E)** Immobile hindlimbs (*n* = 3) have significantly reduced normalized neutrophil (1.0 vs. 0.08, *p* < 0.05) and macrophage counts (1.0 vs. 0.13, *p* < 0.05) compared with mobile hindlimbs (*n* = 3) 1 week after injury; **(F)** H3-Cit immunostaining and DAPI showing NETs in the mobile hindlimb 48 h after injury (40x); **(G)** H3-Cit immunostaining and DAPI showing NETs in the immobile hindlimb 48 h after injury (40x); **(H)** Experimental strategy with DNase I; **(I)** DNase I significantly increases normalized neutrophil (1.0 vs. 6.39, *p* < 0.05) and macrophage (1.0 vs. 3.0, *p* < 0.05) counts in the immobile hindlimb (*n* = 8) 48 h after injury; **(J)** DNase I does not increase normalized neutrophil (1.0 vs. 1.04, *p* = 0.87) and macrophage (1.0 vs. 0.81, *p* = 0.23) counts in the mobile hindlimb (*n* = 5) 48 h after injury; **(K)** H3-Cit immunostaining and DAPI showing NETs in the DNase I-treated immobile hindlimb (40x). All *in vivo* studies had *n* ≥ 3/group. Scale bars are 200 μm. *p < 0.05.

To study how mechanical disruption mediates acute inflammation, we used a model of musculoskeletal trauma with Achilles tendon transection (tenotomy) with a device that provides extremity immobilization. In this model, when mice were allowed to ambulate *ad lib* absent the cast-immobilizer, they develop pathologic ectopic cartilage at the injury site within 3 weeks after injury followed by ectopic bone at the injury site—a condition called heterotopic ossification (29, 30). Mice underwent tenotomy with or without cast-immobilization of the hindlimb (Figure 1A). Histologic examination of the tendon transection site at 48 h and 1 week after injury showed a significant reduction in cellular infiltrates in the immobile hindlimbs (Figures 1B,C, Supplemental Data Figures 1A,B). These findings were confirmed using flow cytometry to quantify neutrophils (CD11b+Ly6G+) and macrophages (CD11b+Ly6G-F4/80+) at the injury site of mobile or immobile hindlimbs (Figures 1D,E, Supplemental Data Figures 3A,B). These findings suggest that immobilization of the tendon transection site reduces acute inflammation. Single cell analysis of the neutrophil population attracted to the injury site 7 days after injury demonstrated early elevation in mRNA encoding several cytokines previously demonstrated to be associated with neutrophil and monocyte attraction and specifically with NETosis (e.g. *Ccl3, Ccl4, Ccr1, Cxcl16, Gpi1, Il1b, Il18, Osm, Tnf*), when compared with non-inflammatory mesenchymal cell populations (Supplemental Data Figures 2A–K). Chemotactic agents derived from non-inflammatory cells, like *Cxcl12*, were found to be relatively down-regulated in infiltrating neutrophils (31–38). Given these data, we examined the presence of NETs in the immobile and mobile hindlimbs 48 h after injury. Citrullinated histones represent one way to visualize NETs (20). Histologic examination showed expansive NETs (H3Cit+DAPI+) in the mobile hindlimb; NETs were present in the immobile hindlimb, but they were substantially reduced in number and expansive character (Figures 1F,G).

To control for potential confounding variables associated with movement in the mobile hindlimb, we examined whether chemical destabilization of NETs in the immobile hindlimb is capable of propagating the inflammatory response. Mice received tenotomy and cast immobilization with intravenous DNase I to enzymatically disrupt the DNA scaffold of NETs (26) (Figure 1H). DNase I has previously been described for transient chemical disruption of NETs with *in vivo* efficacy through systemic administration (28, 39). DNase I significantly increased the number of neutrophils and macrophages at the tendon transection site 48 h after injury in the immobile hindlimb (Figure 1I, Supplemental Data Figure 4). This effect persisted when assessed 1 week after injury as well (Supplemental Data Figures 5A,B). These findings suggest that chemically destabilized NETs augment inflammation. When mobile mice were treated with DNase I, however, treatment did not alter levels of infiltrations of macrophages and neutrophils 48 h after injury suggesting that DNase I and movement may have redundant effects on NETs (Figure 1J, Supplemental Data Figure 6). Immobile hindlimbs in mice treated with DNase I had more expansive NETs when compared with immobile hindlimbs in control-treated mice (Figure 1K).

**Figure 2:**
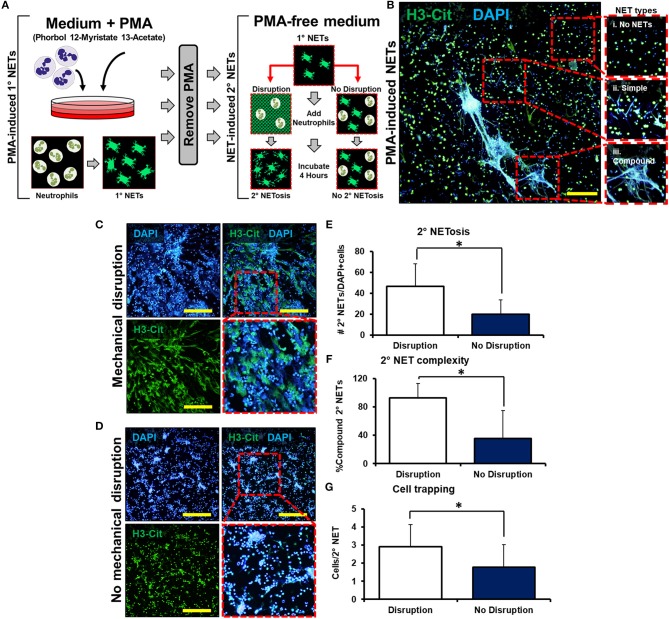
Mechanically disrupted NETs induce NETosis. **(A)** *In vitro* experimental set-up to evaluate whether mechanical disruption of NETs can induce NETosis; **(B)** Representative images from PMA-induced NETs to demonstrate types of NETs; **(C)** 2° NETs with mechanical disruption of 1° NETs; **(D)** 2° NETs without mechanical disruption of 1° NETs; **(E)** Number of 2° NETs normalized to the number of DAPI+ cells per hpf (46.6 vs. 20.2, *p* < 0.05); **(F)** Number of compound 2° NETs normalized to the number of all NETs per hpf (92.7 vs. 35.8, *p* < 0.05); **(G)** Number of DAPI+ nuclei normalized to the number of 2° NETs per hpf (2.9 vs. 1.8, *p* < 0.05). Scale bars are 200 μm. All *in vitro* studies had *n* = 15 hpf/group. *p < 0.05.

We next employed a series of *in vitro* experiments to determine whether mechanically disrupted NETs augment inflammation by inducing NETosis of other neutrophils (Figure 2A). An initial set of mouse-derived neutrophils (1° neutrophils) was exposed to phorbol 12-myristate 13-actetate (PMA) to induce NETosis (1° NETs). Subsequently, the medium was gently exchanged for fresh media *without* PMA. In this new medium, 1° NETs were gently pipetted to induce mechanical disruption (“mobile”), or left intact without pipetting (“immobile”), and a second wave of neutrophils (2° neutrophils) was introduced (Figure 2A). NET-induced NETosis was evaluated using PMA-induced NETs as a guide for quantification and cell trapping (Figure 2B). When 1° NETs were gently disrupted after media change, 2° neutrophils underwent NETosis with expansive 2° NETs (Figure 2C); however, 2° neutrophils did not form similarly expansive structures when 1° NETs were left undisturbed after medium exchange (Figure 2D). These observations were confirmed based on metrics including increased mean number of NETs per high-powered field (hpf) (Figure 2E), increased NET complexity with mechanical disruption (Figure 2F), and increased number of trapped cells per NET (Figure 2G). As expected, in the absence of PMA, 1° neutrophils did not form NETs (Supplemental Data Figure 7A).

To assess that residual PMA present after the media change was not responsible for causing NETosis among 2° neutrophils, a control experiment was performed in which medium with PMA (without 1° neutrophils) was changed and replaced with media followed by addition of 2° neutrophils (Supplemental Data Figure 7B). In this control experiment, 2° neutrophils did not undergo PMA-induced NETosis verifying adequate removal of PMA in this experimental setup (Supplemental Data Figure 7C). Although this allows us to assess the removal of a majority of PMA from the chamber and associated media, it does not account for PMA that may be captured within the 1° NETs themselves. There remains the possibility of residual PMA presence even after media washing, which is subsequently released from the disrupted NETs. Finally, NETs were not observed after disruption of 1° NETs without the addition of 2° neutrophils, indicating that (1) mechanical disruption effectively disaggregates NETs, and (2) NETs observed after the addition of 2° neutrophils were indeed 2° NETs and not re-settled 1° NETs (Supplemental Data Figures 8A,B). These results indicate that mechanically disrupted NETs induce NETosis, a phenomenon which can be described as “NET-induced NETosis.”

**Figure 3:**
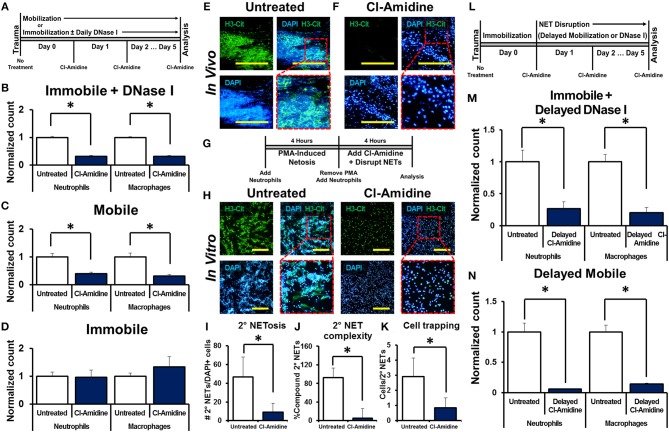
NET-induced NETosis augments inflammation. **(A)** *In vivo* experimental design with Cl-Amidine; **(B)** Cl-Amidine significantly reduces normalized neutrophil (1.0 vs. 0.31, *p* < 0.05) and macrophage (1.0 vs. 0.32, *p* < 0.05) counts in the immobile hindlimb of DNase I-treated mice (*n* = 4) 48 h after injury vs. untreated immobile plus DNase I-treated controls (*n* = 3); **(C)** Cl-Amidine significantly reduces normalized neutrophil (1.0 vs. 0.40, *p* < 0.05) and macrophage (1.0 vs. 0.31, *p* < 0.05) counts in the mobile hindlimb (*n* = 5) 48 h after injury vs. untreated mobile controls (*n* = 5); **(D)** Cl-Amidine does not reduce normalized neutrophil (1.0 vs. 0.96, *p* = 0.79) or macrophage (1.0 vs. 1.35, *p* = 0.08) counts in the immobile hindlimb without DNase I (*n* = 5) 48 h after injury vs. untreated immobilized controls (*n* = 5); **(E)** H3-Cit immunostaining and DAPI showing NETs in the mobile hindlimb 48 h after injury; **(F)** H3-Cit immunostaining and DAPI showing NETs in the mobile hindlimb with Cl-Amidine 48 h after injury; **(G)** *In vitro* experimental set-up with Cl-Amidine; **(H)** H3-Cit and DAPI showing 2° NETs with or without Cl-Amidine after mechanical disruption of 1° NETs; **(I)** Number of 2° NETs normalized to the number of DAPI+ cells per hpf with Cl-Amidine (46.6 vs. 9.1, *p* < 0.05); **(J)** Number of compound 2° NETs normalized to the number of all NETs per hpf with Cl-Amidine (92.7 vs. 5.4, *p* < 0.05); **(K)** Number of DAPI+ nuclei normalized to the number of 2° NETs per hpf with Cl-Amidine (2.9 vs. 0.9, *p* < 0.05); **(L)** *In vivo* experimental-set up with delayed Cl-Amidine; **(M)** Cl-Amidine significantly reduces normalized neutrophil (1.0 vs. 0.27, *p* < 0.05) and macrophage (1.0 vs. 0.20, *p* < 0.05) counts in the immobile hindlimb of DNase I-treated mice 48 h after injury vs. immobile DNase I-treated controls; **(N)** Cl-Amidine significantly reduces normalized neutrophil (1.0 vs. 0.06, *p* < 0.05) and macrophage (1.0 vs. 0.14, *p* < 0.05) counts in the mobile hindlimb 48 h after injury vs. untreated mobile controls. Scale bars are 200 μm. All *in vivo* studies had *n* ≥ 3/group. All *in vitro* studies had *n* = 15 hpf/group. *p < 0.05.

We next examined whether NET-induced NETosis is responsible for the inflammatory response observed in DNase I-treated hindlimbs using Cl-Amidine, an inhibitor of peptidyl arginine deiminase 4 (PAD4) and consequently citrullination which has previously been demonstrated to diminish NETosis (16, 17, 40–45). Mice received tenotomy, cast immobilization, and DNase I with or without immediate Cl-Amidine (Figure 3A). Immediate Cl-Amidine significantly reduced neutrophil and macrophage infiltration in DNase I-treated mice with hindlimb immobilization (Figure 3B, Supplemental Data Figure 9A). These findings demonstrate that the inflammatory effect of DNase I is dependent on the ability of neutrophils to undergo NETosis. A similar reduction in both neutrophil and macrophage infiltration was demonstrated in the mobile hindlimbs of Cl-Amidine-treated mice (Figure 3C, Supplemental Data Figure 9B). Finally, Cl-Amidine did not reduce macrophage or neutrophil recruitment in the immobile hindlimb of mice which did not receive DNase I (Figure 3D, Supplemental Data Figure 9C).

Histologic sections of the mobile hindlimb from Cl-Amidine-treated mice confirmed a reduction in NETs based on immunostaining, when compared with mobile hindlimbs from untreated mice (Figures 3E,F). These findings suggest that NETosis links post-injury inflammation with mechanical strain. However, these findings do not establish that disrupted NETs augment inflammation, as Cl-Amidine administered immediately after injury prevented initial NET formation (Figures 3E,F). Given the reduction in citrullination (H3-Cit) associated with PAD inhibition *in vivo* we next sought to identify whether other markers of neutrophil activity, specifically myeloperoxidase (MPO) would demonstrate the same results. We found that MPO appropriately costained with H3-Cit and DAPI in NETs formed *in vivo* (Supplemental Data Figure 10). Under conditions of Cl-Amidine treatment there was a gross reduction in formation of MPO (+) NETs (Supplemental Data Figure 10).

Therefore, we first confirmed that Cl-Amidine inhibits NET-induced NETosis using a series of *in vitro* experiments. The addition of Cl-Amidine to 2° neutrophils prevented 2° NETosis caused by mechanically disrupted 1° NETs (Figures 3G,H). These observations were confirmed based on metrics including increased mean number of NETs per hpf (Figure 3I), increased NET complexity with mechanical disruption (Figure 3J), and increased number of trapped cells per NET (Figure 3K). To demonstrate that Cl-Amidine prevents NET-induced NETosis *in vivo*, mice received tenotomy and were initially immobilized to allow NETosis to proceed, followed by chemical or mechanical disruption and simultaneous Cl-Amidine 24 h after injury (Figure 3L). Histologic sections from immobile hindlimbs taken 24 h after injury confirmed the presence of NETs (Supplemental Data Figure 11). Immobile hindlimbs from mice treated with delayed DNase I and Cl-Amidine 24 h after injury had significantly reduced neutrophil and macrophage counts when compared with mice that received DNase I, but not Cl-Amidine 24 h after injury (Figure 3M, Supplemental Data Figure 12A). Similarly, hindlimbs which were allowed to be mobile and treated with Cl-Amidine after 24 h of post-injury immobilization had significantly reduced neutrophil and macrophage counts when compared with hindlimbs that were allowed to be mobile 24 h after injury without Cl-Amidine (Figure 3N, Supplemental Data Figure 12B).

**Figure 4:**
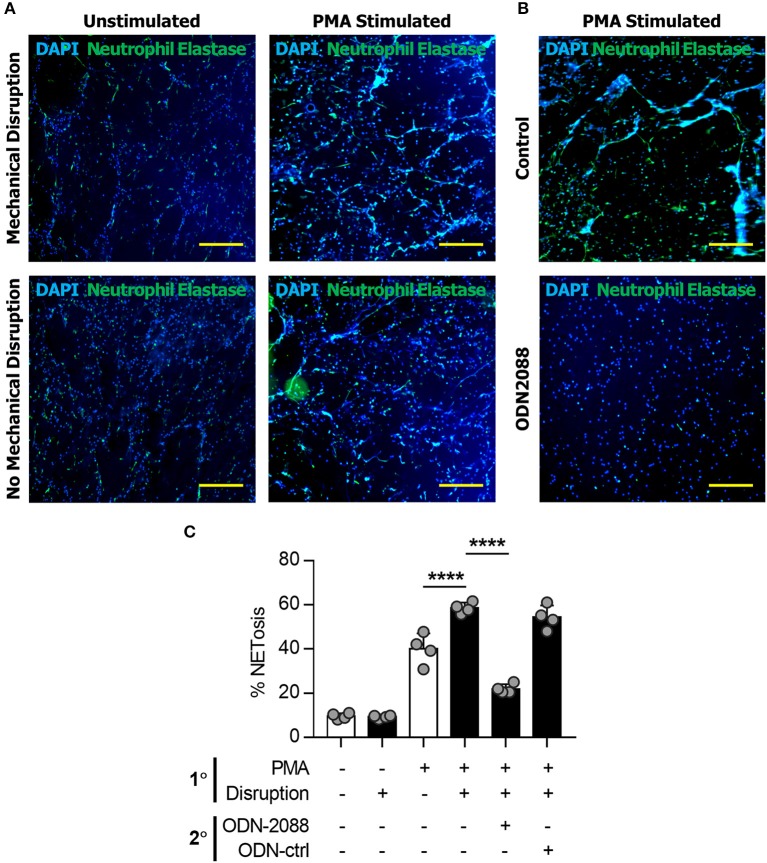
NETs induce NETosis in human neutrophils. **(A)** Human neutrophils *in vitro* demonstrating the increase in NET formation after Mechanical Disruption of primary (PMA-induced) NETs. **(B)** Demonstration of the effect of ODN2088 on NET-induced NETosis of human neutrophils *in vitro*. **(C)** Quantification of increased NETosis after disruption of primary NETs; this effect is abrogated by ODN2088. Scale bars are 300 μm. ****p < 0.0001, by one-way ANOVA.

Neutrophils in mice have several physiologic differences from those found in humans and can have significantly different responses to stress, infection, and the inflammatory response. To determine whether this was a model specific phenomenon we next isolated human neutrophils which were evaluated *in vitro* utilizing the same techniques previously applied to murine cells. Human-derived neutrophils demonstrated similar NET-forming behavior to murine cells (Figure 4A). Human-derived neutrophils demonstrated evidence of NET-induced NETosis *in vitro (*Figures 4A,C).

**Figure 5:**
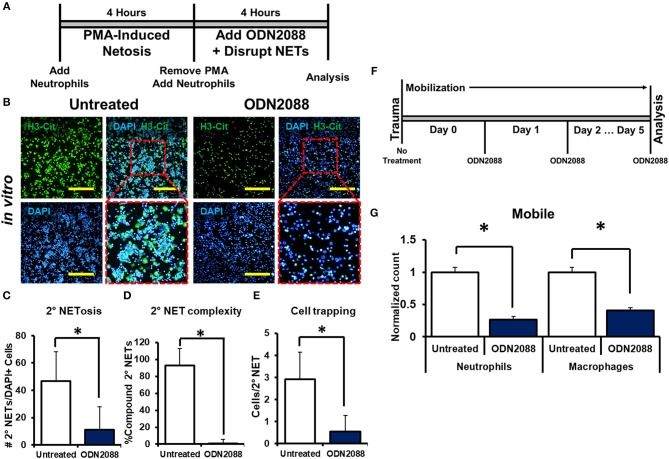
NETs induce NETosis through TLR activity. **(A)** *In vitro* experimental design with ODN-2088; **(B)** H3-Cit and DAPI showing 2° NETs with or without ODN-2088 after mechanical disruption of 1° NETs; **(C)** Number of 2° NETs normalized to the number of DAPI+ cells per hpf with ODN-2088 (46.6 vs. 11.0, *p* < 0.05); **(D)** Number of compound 2° NETs normalized to the number of all NETs per hpf with ODN-2088 (92.7 vs. 0.90, *p* < 0.05); **(E)** Number of DAPI+ nuclei normalized to the number of 2° NETs per hpf with ODN-2088 (2.9 vs. 0.60, *p* < 0.05); **(F)** *In vivo* experimental set-up with ODN-2088; **(G)** ODN-2088 significantly reduces normalized neutrophil (1.0 vs. 0.27, *p* < 0.05) and macrophage (1.0 vs. 0.41, *p* < 0.05) counts in the mobile hindlimb 48 h after injury vs. untreated mobile controls; All *in vivo* studies had *n* ≥ 3/group. All *in vitro* studies had *n* = 15 hpf/group. Scale bars are 200 μm. *p < 0.05.

We next sought to determine *the mechanism through which* disrupted NETs induce NETosis. Because DNA is a substantial component of NETs, we hypothesized that toll-like receptors (TLRs) 7/8/9, known receptors for oligonucleotides, are mediators of NET-induced NETosis (46–52). In addition, NETs have been shown to activate dendritic cells and monocytes through TLR activity. *in vitro* treatment with ODN-2088, an oligonucleotide inhibitor of TLR 7/8/9, significantly reduced 2° NETosis after mechanical disruption of 1° NETs (Figures 5A,B). These observations were confirmed quantitatively (Figures 5C–E). However, ODN-2088 did not inhibit PMA-induced NETosis, indicating that non-TLR-mediated NETosis remained intact despite ODN-2088 (Supplemental Data Figures 13A,B). The mobile hindlimbs of mice treated with ODN-2088 showed significantly reduced macrophage and neutrophil infiltration (Figures 5F,G, Supplemental Data Figure 14). Likewise, the response of human neutrophils to ODN2088 during NETosis matched what we had previously identified in murine cells (Figures 4B,C).

## Discussion

The interaction between movement or mechanical strain and inflammation after trauma carries implications for the care of the tens of millions of patients with orthopedic and soft tissue injuries annually. NET formation, or NETosis, represents an emerging and critically important field of study in the modulation and amplification of the inflammatory cascade after injury and infection.

Our findings suggest a model in which NETosis is propagated and augmented by the physical or chemical disruption of formed NETs both *in vivo* and *in vitro*. The model of musculoskeletal injury we employed in this study allowed for interrogation of the relationship between mechanical strain and inflammation because the hindlimb can be immobilized. Other models in which mechanical strain can induce inflammatory responses such as ventilator-associated lung injury or reperfusion injury do not provide a similar opportunity for “immobilization.” If a link were to exist between mechanical fragmentation of NETs and propagation of NETosis then we would expected that animals which had been immobilized would demonstrate a decrease in both neutrophil migration to the site of injury and in the size, number, and complexity of NET structures *in vivo* when compared with those which had been allowed to ambulate post-trauma.

While our data supported this hypothesis, the degree of confounding factors associated with mobile vs. immobilized extremities rendered it difficult to draw conclusions from these experiments in isolation. Thus, we sought to identify a non-mechanical surrogate mechanism to confirm our findings. DNase I, when provided exogenously, acts to facilitate the enzymatic breakdown of extracellular DNA thus chemically disrupting a critical part of the backbone of a NET and leading to the breakdown of the NET structure. As DNase I should cleave NETs by a fundamentally different mechanism when compared with mechanical strain, we hypothesized that an immobilized mouse should show some evidence of NET disruption and consequently enhanced inflammation with DNase I administration. Furthermore, as the half-life of DNase I *in vivo* is short, greatest effect should occur upon the primary NETs, while, at least partially, sparing secondary NETs which form later. Again, our data supported evidence of NET disruption followed by enhanced inflammation with DNase I administration, so we next asked how the combination of mechanical and chemical disruption would affect the inflammatory cascade after injury. Interestingly, we found that addition of DNase I to mobilized animals did not augment the inflammatory response to the degree found in immobilized animals. While unexpected, this would be consistent with a model in which both mobility and DNase I are acting by a similar downstream mediator without affecting separate or confounding parts of the inflammatory cascade.

Our *in vitro* findings suggest that NETs are susceptible to mechanical disruption, leading to gross disintegration of their superstructure. Furthermore, when NETs are mechanically disrupted there is a more robust NET-forming response in new neutrophils added to the system compared to when these 1° NETs are kept intact. These data suggest a link between the presence of mechanically disrupted NETs and NETosis in otherwise inactive neutrophils—a process we refer to as NET-induced or 2° NETosis. Given the link implied by our *in vitro* findings between mechanical strain and the propagation of NETosis, we sought to identify whether movement may be sufficient to physical disrupt the fragile structure of NETs after injury and induce the NET-induced NETosis response.

Based upon our *in vitro* data, we hypothesized that disruption of the NET superstructure and the presence of NET fragments may represent the central signaling agent mediating this enhanced inflammatory response. However, there remained the possibility that, while DNase I and mechanical strain shared a single mechanism in their effect upon neutrophil infiltration, that mechanism could still be something other than NETosis. To address this we utilized Cl-Amidine, a PAD4 inhibitor which had previously been demonstrated to prevent to formation of NETs *in vivo*. We hypothesized that introduction of Cl-Amidine to the system would render it resistant to perturbations in inflammation secondary to any mechanism which specifically targeted NETosis. Cl-Amidine resulted in diminished inflammatory infiltrate in both the mobile untreated and immobile, DNase-treated animal, however, it did not further attenuate the inflammatory response present in the immobilized animal. This was consistent both with immediate administration of the PAD4 inhibitor and delayed treatment suggesting that the action of mechanical stress and exogenous DNase I is inhibited specifically by inhibition of NETosis even in a system where NETs had previously been formed.

Given the above findings, we are led to believe in the following five points: (1) Addition of neutrophils to a system of previously disrupted NETs will result in more robust NETosis when compared with a system of in which the initial NETs were not disrupted; (2) Mechanical stress and exogenous DNase I both increase post-injury neutrophil infiltration; (3) Mechanical stress and exogenous DNase I share a common downstream signaling mediator; (4) The increase in post-injury neutrophil infiltration is dependent upon the presence of NETs; and (5) The action of mechanical stress and exogenous DNase I is inhibited by inhibition of NETosis even in a system where NETs have previously formed. Based upon the above, we propose a model of NET-induced NETosis whereby mechanically disrupted NETs augment NETosis, NETosis augments inflammation, and that this is a mechanism which links mechanical strain to inflammation (Supplemental Data Figure 15).

We then utilized this framework to try and better identify the pathway by which NET-induced NETosis modulated the inflammatory cascade. Previous studies have shown that NETs can activate monocytes and dendritic cells through TLRs. However, direct propagation of the inflammatory response through TLR-mediated, NET-induced NETosis would represent an important advance in our understanding of how movement and mechanical strain augment inflammation. We utilized both our *in vitro* and *in vivo* models to demonstrate that ODN-2088 inhibition of TLR 7/8/9 significantly reduced NET-induced but not PMA-induced NETosis. Consistent with our findings, previous studies have shown that NETs contain mitochondrial DNA (mtDNA) while others have shown that mtDNA released from damaged tissue after trauma induce NETosis (53). In the context of our findings, post-injury release of mtDNA from endogenous tissues may be the cause for *initial* NETosis as seen in the immobile hindlimb; however propagation of the inflammatory response caused by movement is likely due to disrupted NETs, which are not immediately cleared from the injury site. Our findings also identify a novel mechanism through TLR inhibitors may reduce inflammation, specifically by preventing NET-induced NETosis. Furthermore TLR 7/8/9, which are inhibited by ODN-2088, are oligonucleotide receptors suggesting that NET-induced NETosis is mediated through the DNA component of disrupted NETs. The specificity of these pathways remains an area for future study to identify which specific TLRs are associated with this phenomenon and whether this link might be utilized for therapeutic benefit moving forward.

Our findings shed light on a poorly understood clinical phenomenon—the exacerbation of inflammation with movement or mechanical strain. Neutrophil activity represents one of the earliest responses to injury. While movement and destabilized NETs may activate other cells including monocytes or dendritic cells, our findings suggest that the earliest propagation of inflammation is likely fueled by neutrophils activated by disrupted NETs. The identification of TLR-mediated NET-induced NETosis presents targets to mitigate inflammation, which will benefit patients who are unavoidably faced with mechanical strain involving sites of tissue damage such as those with musculoskeletal injury, joint arthropathy, ventilator-induced lung injury, and reperfusion injury (13–15).

## Materials and Methods

### Ethics Statements

This study was carried out in accordance with the recommendations of the University of Michigan's Human Research Protection Program (HRPP). The protocol (HUM00044257) was approved by the Institutional Review Boards of the University of Michigan Medical School (IRBMED). All subjects gave written informed consent in accordance with the Declaration of Helsinki. Additionally, this study was carried out in accordance with the guidelines provided in the Guide for the Use and Care of Laboratory Animals from the Institute for Laboratory Animal Research (ILAR, 2011) and were approved by the Institutional Animal Care and Use Committee of the University of Michigan (IACUC) under protocol (PRO0005909).

### Animal Housing

All animals were housed in UCUCA-supervised facilities, not to exceed four mice housed per cage at 72 ± 4°F, receiving 12 h of light exposure each day, with no diet restrictions. For all *in vitro* and *in vivo* studies, 8–10 weeks old, mixed-gender, C57BL/6J mice from Charles River (Wilmington, MA) were defined as wild type.

### Mouse Cell Isolation

Neutrophils were isolated from bone marrow collected from the femurs of wild type C57BL/6 mice using layered Histopaque gradient (Sigma-Aldrich) isolation as previously described (53). Neutrophils (1° neutrophils) were seeded at 2 × 10^4^ cells in 500 μl RPMI 1640 (Corning) + 2% fetal bovine serum (FBS, Corning) per well to an 8-well chamber slide and incubated for 1 h at 37°C under 5% CO_2_.

### Human Cell Isolation

Human neutrophils were isolated as we have done previously (54). Blood from healthy volunteers was collected by venipuncture into heparin tubes, and fractionated by Ficoll/Hypaque gradient (GE Healthcare) to separate peripheral-blood mononuclear cells from neutrophils. Neutrophils were then further purified by dextran sedimentation of the red blood cell layer, before lysing residual red blood cells with 0.2% sodium chloride.

### Generation of 1° Mouse NETs *in vitro*

Seeded 1° neutrophils were stimulated by the addition of 100 μL of 600 nM Phorbol 12-myristate 13-acetate (PMA, Sigma-Aldrich) in RPMI (55–58). Unstimulated control well-received 100 μL RPMI without PMA. Treatment control groups additionally received either ODN-2088 (10 μm, Invivogen) or Cl-Amidine (200 μm, EMD-Millipore). All samples were then incubated for 4 h at 37°C under 5% CO_2_. Control samples with terminal endpoints at 4 h were quenched via removal of PMA-containing medium and fixed with 10% buffered formalin for 15 min at 4°C in preparation for immunocytochemistry. All experiments were performed in triplicate.

### Generation of 2° Mouse NETs *in vitro*

After incubation PMA-containing medium was removed and fresh PMA-free RPMI + 2% FBS was added. To simulate mechanical disruption of PMA-induced NETs, gentle pipette disruption was performed for 15 s in each well at a rate of ~1 complete plunge and release of the pipette per second for an average of 15 plunges per cycle. A second group of 2 × 10^4^ neutrophils (2° neutrophils) obtained from WT mice were added to wells with or without mechanical disruption and incubated for 4 h. Primary neutrophil only controls in the presence or absence of mechanical disruption were similarly maintained for an additional 4 h in PMA-free media without the addition of 2° neutrophils. At the time of 2° neutrophil addition treatment groups additionally received either ODN-2088 (10 μm) or Cl-Amidine (200 μm). After 4 h all samples were quenched via removal of medium and fixed with 10% buffered formalin for 15 min at 4°C in preparation for immunocytochemistry. All experiments were performed in triplicate.

### Generation of 1° Human NETs *in vitro*

Purified neutrophils were resuspended in RPMI media (Gibco) supplemented with L-glutamine and 2% fetal bovine serum (Gibco). 1 × 10^5^ neutrophils were seeded into each well of poly-L-lysine (Sigma)-coated four-chamber slides (Lab-Tek). To induce NET formation, neutrophils were incubated in the presence of 100 nM PMA (Sigma) for 3 h at 37°C and 5% CO_2_. Following incubation, PMA-containing media was replaced with fresh culture media. Cells were then either left undisrupted or gently pipetted for 15 s per well to simulate mechanical disruption.

### Generation of 2° Human NETs *in vitro*

An additional round of 1 × 105 neutrophils in culture media was added to each well containing primary NETs as described above. Neutrophils were further incubated in the presence of ODN-2088 or ODN-Control (final concentration of 10 μM) for an additional 3 h at 37°C and 5% CO_2_. Following incubation, media was discarded, and cells were fixed with ice-cold 4% paraformaldehyde for 15 min at room temperature, followed by overnight blocking at 4°C in 10% fetal bovine serum diluted in PBS (blocking buffer).

### Tenotomy Model

C57BL/6 mice underwent sterile dorsal hindlimb tendon transection at the midpoint of the Achilles tendon (Achilles' tenotomy) with placement of a single 5-0 vicryl suture to close the skin. Mice were then divided into the following groups for further intervention: mobile, delayed mobile, immobile. These groups were then further subdivided on the basis of treatment with chemical modulators of NETosis: untreated, DNase I treated, Cl-Amidine treated, or ODN-2088 treated. All subgroups had *n* ≥ 3.

### Immobilization

Immediately after injury, mice designated as immobile were fitted with a rigid cast immobilizer over their injured limb. Immobilizers were made from Eppendorf tubes with foam; the immobilizer was placed around the hindlimb and affixed with glue. Rigid immobilization in this manner has previously been described (59). Mice designated as immobile were returned to their cage immediately after placement of the immobilizer. Mice designated as delayed mobile were returned to their cage for 24 h before being anesthetized for removal of their immobilizer. Mice designated as mobile were returned to their cages without placement of an immobilizer. After being returned to their cages groups were allowed to move *ad libitum* until euthanization.

### DNase I Treatment

Mice treated with DNase I received 1,000 units in PBS delivered through intraperitoneal injection (1,000 U/500 μL). Within the DNase I treated groups subjects were divided on the basis of immediate or delayed DNase I injection. Mice receiving immediate DNase I injection were treated immediately after injury and/or the placement of the immobilizer. Mice receiving delayed DNase I injection were treated immediately after removal of their immobilizer 24 h post injury. All DNase I treatments occurred daily after initial injection lasting until terminal sacrifice of the animal. Groups acting as untreated controls for DNase I treatment groups received 500 μL of PBS vehicle only.

### Cl-Amidine Treatment

Mice treated with Cl-Amidine received 0.2 mg in PBS delivered through intraperitoneal injection (0.2 mg/500 μL). Within the Cl-Amidine treated groups subjects were divided on the basis of immediate or delayed Cl-Amidine injection. Mice receiving immediate Cl-Amidine injection were treated immediately after injury and/or the placement of the immobilizer. Mice receiving delayed Cl-Amidine injection were treated immediately after removal of their immobilizer 24 h post injury or at the time of DNase I injection 24 h after injury. All Cl-Amidine treatments occurred daily after initial injection lasting until terminal sacrifice of the animal. Groups acting as untreated controls for Cl-Amidine treatment groups received 500 μL of PBS vehicle only.

### ODN-2088 Treatment

Mice treated with ODN-2088 received 50 μg in PBS delivered through intraperitoneal injection (50 μg/500 μL). All ODN-2088 treatments occurred daily after initial injection lasting until terminal sacrifice of the animal. Groups acting as untreated controls for ODN-2088 treatment groups received 500 μL of PBS vehicle only.

### Tissue Isolation and Digestion

Tissue was isolated from the tendon transection site using a standardized technique. Specifically tissue from the calcaneus (excluding bone) to the convergence of tendon with calf musculature was excised, minced at 4°C and digested. Tissue was digested for 120 min in 2 mg/ml Collagenase 3 (Worthington) in Hanks Balanced Salt Solution (HBSS) at 37°C under constant agitation. Samples were filtered using a 70-micron sterile strainer and centrifuged at 800 rpm for 5 min before removing the supernatant and washing in HBSS. This process was repeated three times before incubation with fluorescently labeled antibodies.

### Flow Cytometry

Flow cytometry was performed to quantify the number of neutrophils (CD11b+Ly6G+) and macrophages (CD11b+F4/80+Ly6G–) in each sample. Antibodies used included: Ly6G-APC (Clone: 1A8, BD Bioscience; Cat. #: 560599), CD11b-V450 (Clone: M1/70, BD Bioscience; Cat. #: 560455), F4/80-PECy7 (Clone: BM8, eBioscience; Cat. #: 25-4801-82). No Fc block utilized. Following 1 h of incubation at 4°C, sample were washed and filtered through a 45-micron mesh filter before being run on a FACSAria II (BD Biosciences) Cell Sorter at the University of Michigan Flow Cytometry Core in the Biomedical Science Research Center. Samples were gated to separate debris and autofluorescent signals from the cell population. Data were then analyzed using the FlowJo software (TreeStar). Flow cytometric data was normalized to account for differences in aggregate number between cell types. For untreated group flow data was normalized to the mobile sample population. For all experiments with treated and untreated samples values were normalized to the untreated population.

### Histologic Processing and Analyses

At 48 h and 1 week post-injury animals were euthanized for histology. The distal hindlimb was removed by sharp dissection at the hip. Skin was removed carefully to leave the injury site undisturbed and the toes were removed to facilitate rapid decalcification. Decalcification of the sample was completed with 19% ethylenediaminetetracetic acid (EDTA) solution for 28 days at 4°C. Decalcified tissues were dehydrated through graded ethanol, and paraffin embedded. Transverse sections from all sampled were completed with a width of 5 microns and mounted on charged microscope slides (Globe Scientific). Paraffin samples were dried overnight at 37°C. A representative subset of samples were stained for routine H&E to confirm anatomy and identify gross areas of inflammation.

### Immunohistochemistry

Immunofluorescent staining was performed as previously described for Anti-Histone H3 (citrulline R2 + R8 + R17, Abcam; Cat. #: ab5103). Briefly, sections were deparaffinized and rehydrated in xylenes and graded ethanol. Antigen retrieval was performed with Citrate solution pH 6.0. Samples were then quenched for autofluorescence in 3% glycine before blocking and permeabilization. Primary antibodies were applied overnight at 4°C. Appropriate dilutions were determined prior to achieving final images. After washing, fluorescently conjugated secondary antibodies tagged with donkey anti-Rabbit IgG AlexaFluor 488 (Thermo Scientific; Cat. #: A-11008). Nuclear counterstain was DNA dye Hoechst 33342 (Thermo Scientific; Cat. #: 62249) and samples were mounted with aqueous mounting media (Sigma-Aldrich). Primary antibody, secondary antibody, and autofluorescent controls were run simultaneously with each tested sample.

### Quantification of Mouse NETs by Immunocytochemistry

After fixation, *in vitro* samples were gently washed 3 times with phosphate buffered saline (PBS, Thermo Scientific). Primary antibodies used for immunostaining included Anti-Histone H3 (citrulline R2 + R8 + R17, Abcam; Cat. #: ab5103). DNA was stained by the DNA dye Hoechst 33342 (Thermo Scientific; Cat. #: 62249). Secondary antibodies used included donkey anti-Rabbit IgG AlexaFluor 488 (Thermo Scientific; Cat. #: A-11008). After staining samples prepared with aqueous mounting media (Sigma-Aldrich) and cover-slipped for microscopy.

### Quantification of Human NETs by Immunocytochemistry

NETs were detected by incubating with anti-human neutrophil elastase antibody (Abcam) for 1 h at 4°C, followed by incubation with FITC-conjugated secondary antibody (Southern Biotech) for an additional hour at 4°C. Nuclear DNA was stained with Hoechst 33342 (Invitrogen) for 10 min at room temperature. The chamber was gently removed from the attached slide, and ProLong Gold Antifade reagent (Invitrogen) was applied directly to the cells. The mount was secured in place by carefully lowering a coverslip onto the slide. Images were collected with a Cytation 5 Cell Imaging Multi-Mode Reader (BioTek). The percentage of NETs (decondensed extracellular DNA co-localized with neutrophil elastase) was determined as the average of six to eight fields (20x). Experiments were performed at least four times (independent biological replicates).

### Microscopy

All tissue sections and fluorescently stained samples were imaged using an Olympus BX-51 upright light microscope equipped with standard DAPI, 488, and TRITC reflector cubes attached to an Olympus DP-70 high resolution digital camera. H&E stained sections were imaged at 4 and 10x magnification. H3-Cit stained tissue sections were imaged at 40x magnification. All *in vitro* samples were imaged at 10 or 20x magnification. Scale bars were placed for all images with a standard 200 μm diameter. Images were visualized in Adobe Photoshop to perform to perform quantification of NET behavior and to overlay cells for co-staining.

### Quantification of *in vitro* NETosis Assays

All *in vitro* experiments were performed in technical triplicates with 5 high-power fields evaluated per well (total *n* = 15 per tested group). High-power fields (hpfs) were selected using a random number generator and NETs quantified by two separate blinded observers with specific training for identification and categorization of NETs. For the purpose of *in vitro* quantification we defined a NET as: (1) a DAPI+/H3–Cit+ co-staining structure extending beyond a distinct nucleus with (2) loss of nuclear lobulation, and (3) one or more of the following: (a) ≥1 distinct branching structure (b) evidence of complete cytoplasmic disruption/rupture. Secondary NETosis is quantified as the average number of NETs as a fraction of distinct DAPI+ nuclei in each hpf. Secondary NET complexity is quantified as the fraction of NETs which exhibit one or more of the following properties: (a) >3 distinct branching structures, (b) evidence of complete cytoplasmic disruption/rupture. As a third metric we examined the ability of 2° NETs to trap other neutrophils. We defined cell trapping as the average number of distinct DAPI+ nuclei either (a) within or (b) immediately contiguous to a NET.

### Single Cell Analysis

Sequencing data were first pre-processed using the 10X Genomics software Cell Ranger (10x Genomics Inc., Pleasanton, CA, USA) and aligned to mm10 genome. Cells were collected from 15 samples for four time points (4 samples for days 0, 7, and 21; 3 samples for day 3). Our time points of interest are days 3 and 7, corresponding to the peak of the inflammation. Downstream analysis steps were performed using Seurat. For each time point, cells with fewer than 500 genes per cell or more than 60,000 UMIs, and genes expressed in fewer than 10 cell were filtered out for quality control. The downstream analysis steps for each sample type include normalization, scaling, calculation of the variable genes, canonical correlation analysis, dimensionality reduction (t-SNE), unsupervised clustering, and the discovery of differentially expressed cell-type specific markers. Eight clusters were defined by unsupervised Louvain algorithm applied to the first 10 aligned canonical components. Violin plots of known markers were utilized to identify a mixed inflammatory/myeloid cluster (11,565 cells). Cells from other clusters were filtered out. We repeated the procedure, followed the method described above and found 9 sublcusters for the inflammatory/mieloyd cluster. To extract the neutrophils, firstly we characterize the subclusters we used Immgen [The Immunological Genome Project: network of gene expression in immune cells (60)]. Two subclusters (total 2,923 cells) were enriched for at two or three out of three granulocyte populations. Secondly, cells belonging to the two granulocyte-neriched subclusters were labeled as neutrophils if they were they showed a normalized expression of both Ccr1 and Csf3r 0.5 > 0. This conditions retrieved 602 and 85 neutrophils at days for days 3 and 7.

### Power Analysis and Allocation

Animal allocation was performed as described above for specific treatment groups. *A priori e*xclusion criteria for animals used in the experiments included only specimens requiring early termination of experiment for humane reasons—no animals necessitated exclusion from final statistical analysis. Animals were not formally randomized to treatment groups. Prior to animal allocation a power analyses were performed for each individual outcomes of interest. For flow cytometric analyses our outcome of interest was difference in aggregate number or neutrophils or macrophages as defined above. To confirm a 50% decrease in cell population with a power of 0.8 assuming a standard deviation of 8,000 cells and a mean of 40,000 cells in untreated mice, we required 3 mice per group. Consequently, all flow cytometric experiments were performed with *n* ≥ 3 animal per group.

### Statistical Analysis

All statistical analyses were performed using Student's *t*-test analysis with sample size *n* ≥ 3 for all groups. Statistical analysis was performed using an appropriate analysis of variance when more than two groups were compared, followed by a *post hoc* Student's *t*-test to directly compare two groups. Inequality of standard deviations was excluded by using the Levene's test. Outlier analysis for was performed using Grubbs' test for outliers with an alpha of 0.05. Flow cytometric data was normalized as described above. In figures, bar graphs represent means, whereas error bars represent one standard deviation. Asterisks are representative of statistical significance. Statistical significance was considered for *p* < 0.05.

## Ethics Statement

This study was carried out in accordance with the recommendations of the University of Michigan's Human Research Protection Program (HRPP). The protocol (HUM00044257) was approved by the Institutional Review Boards of the University of Michigan Medical School (IRBMED). All subjects gave written informed consent in accordance with the Declaration of Helsinki. Additionally, this study was carried out in accordance with the guidelines provided in the Guide for the Use and Care of Laboratory Animals from the Institute for Laboratory Animal Research (ILAR, 2011) and were approved by the Institutional Animal Care and Use Committee of the University of Michigan (IACUC) under protocol (PRO0005909).

## Author Contributions

SA, SJL, and BL contributed to the conception and design of the study. SA, SJL, DC, JL, GB, and SL acquired data. SA, SJL, SL, WC, MD, TS, SK, YM, PW, SM, and BL analyzed and provided critical interpretation of data. SA wrote the first draft of the manuscript. SA, SJL, SL, MD, YM, and BL wrote sections of the manuscript and/or designed figures. SA, SJL, SL, WC, MD, TS, SK, YM, PW, and BL provided critical review and edited the manuscript and/or figures. All authors contributed to manuscript revision, read, and approved the submitted version.

### Conflict of Interest Statement

The authors declare that the research was conducted in the absence of any commercial or financial relationships that could be construed as a potential conflict of interest.
