# Supplementary material for: Disruption of Neutrophil Extracellular Traps (NETs) Links Mechanical Strain to Post-traumatic Inflammation
Source: Front Immunol. 2019 Oct 24;10:2148. doi: 10.3389/fimmu.2019.02148 (PMC6821718; doi:10.3389/fimmu.2019.02148)
Supplement: Supplementary file 1 [file Data_Sheet_1.PDF]

SUPPLEMENTAL INFORMATION

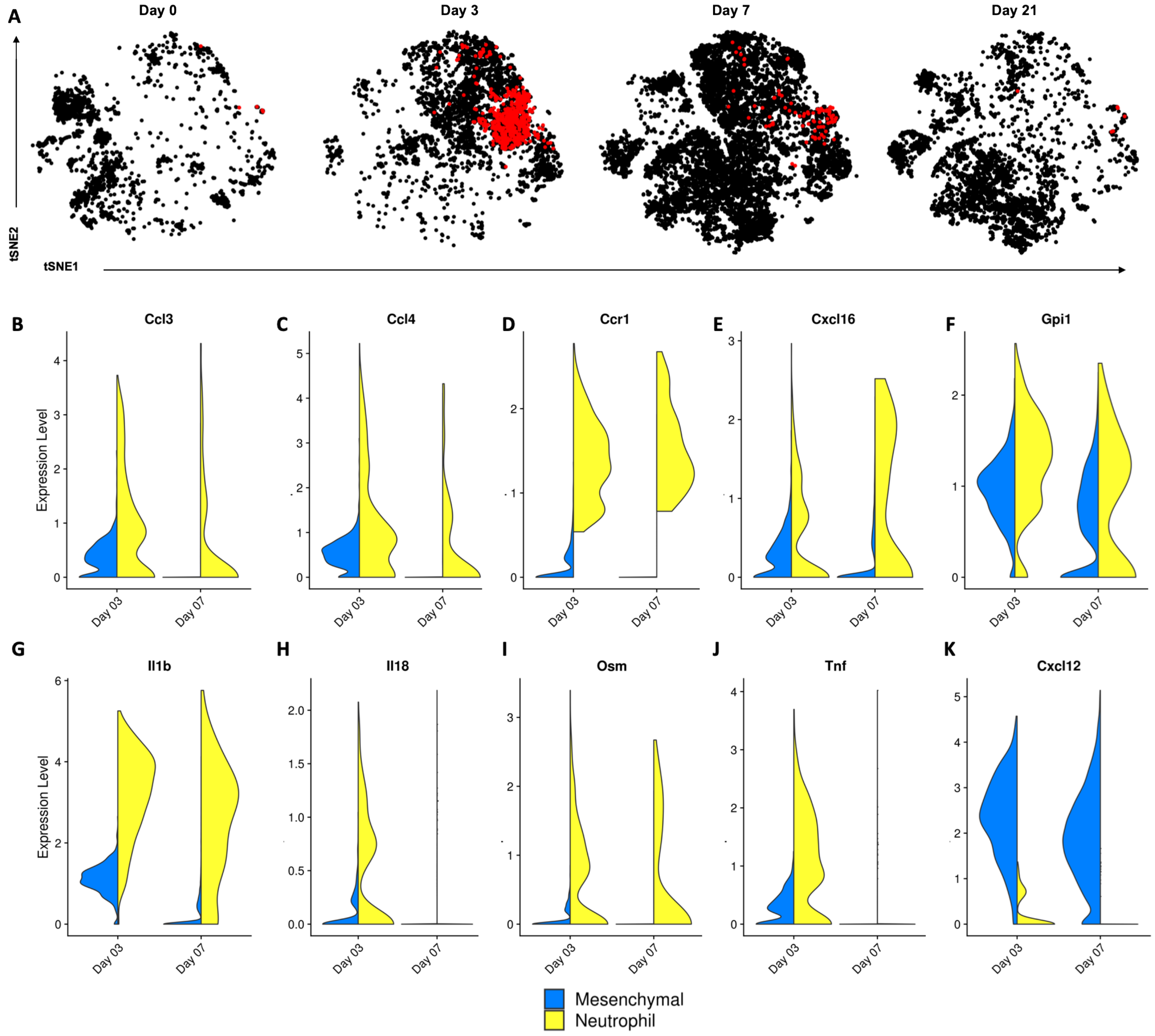

Supplemental Data Fig 1: RNA sequencing demonstrates a pro-inflammatory phenotype consistent with TLR activation. A) Canonical analysis across multiple timepoints (Day 0, 3, 7, 21) demonstrating a heterogenous set of populations at the tendon and site of injury. Total n=3-4/timepoint. Isolation of neutrophils (red) by transcriptome demonstrates parallel patterns of acute peak at day 3 and resolution by day 21 (B-J). Expression levels of well characterized inflammatory gene signaling in neutrophils with mesenchymal cells for comparison subgroup. Timepoints reflect the window where neutrophils are found at the site of injury. K) Negative control gene well characterized as involved in neutrophil recruitment but secreted by non-neutrophilic sources as internal control.

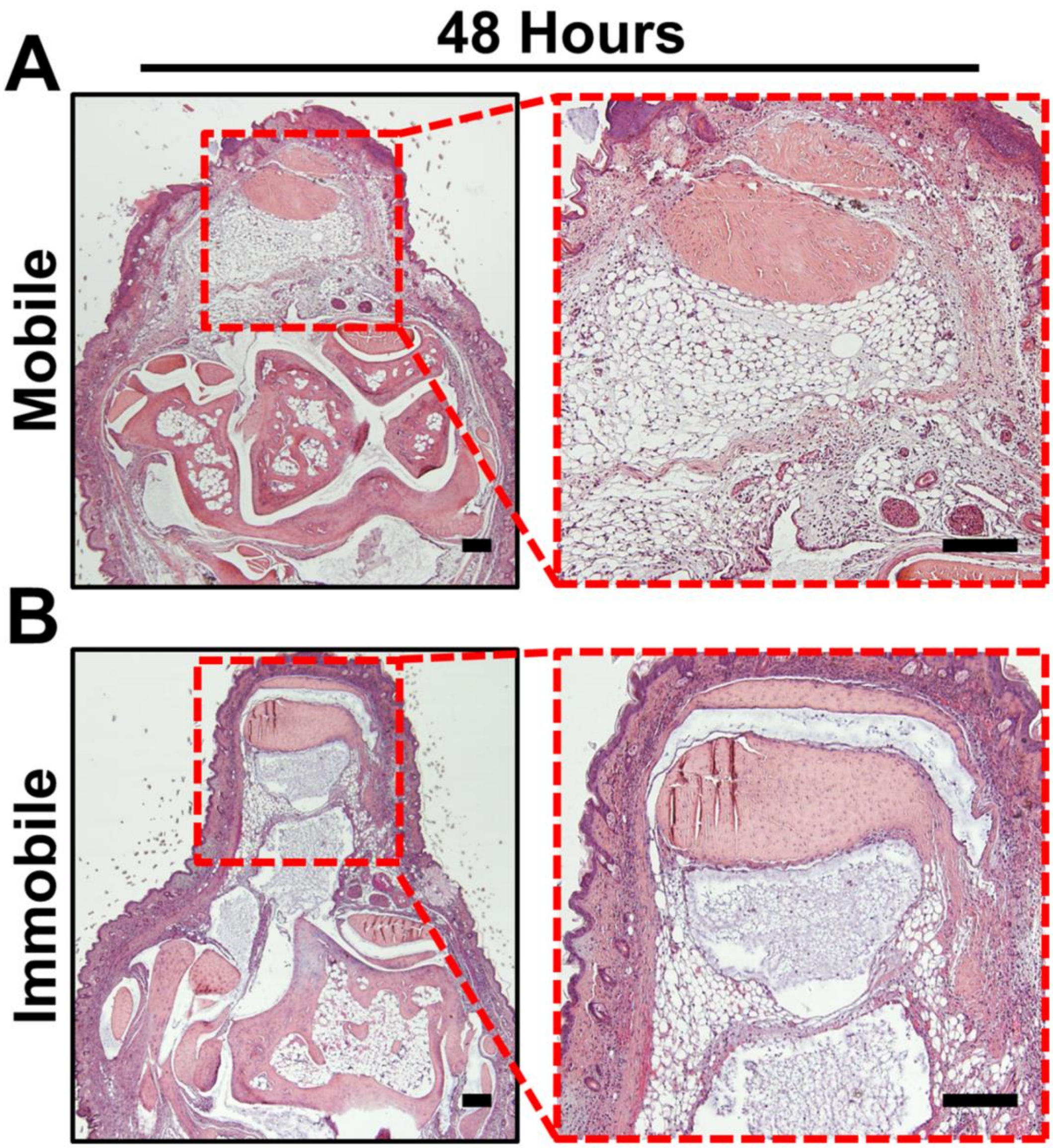

Supplemental Data Fig 2. (A) H&E of mobile hindlimb 48 hours after injury; (B) H&E of immobile 48 hours after injury. Scale bars are 200µm.

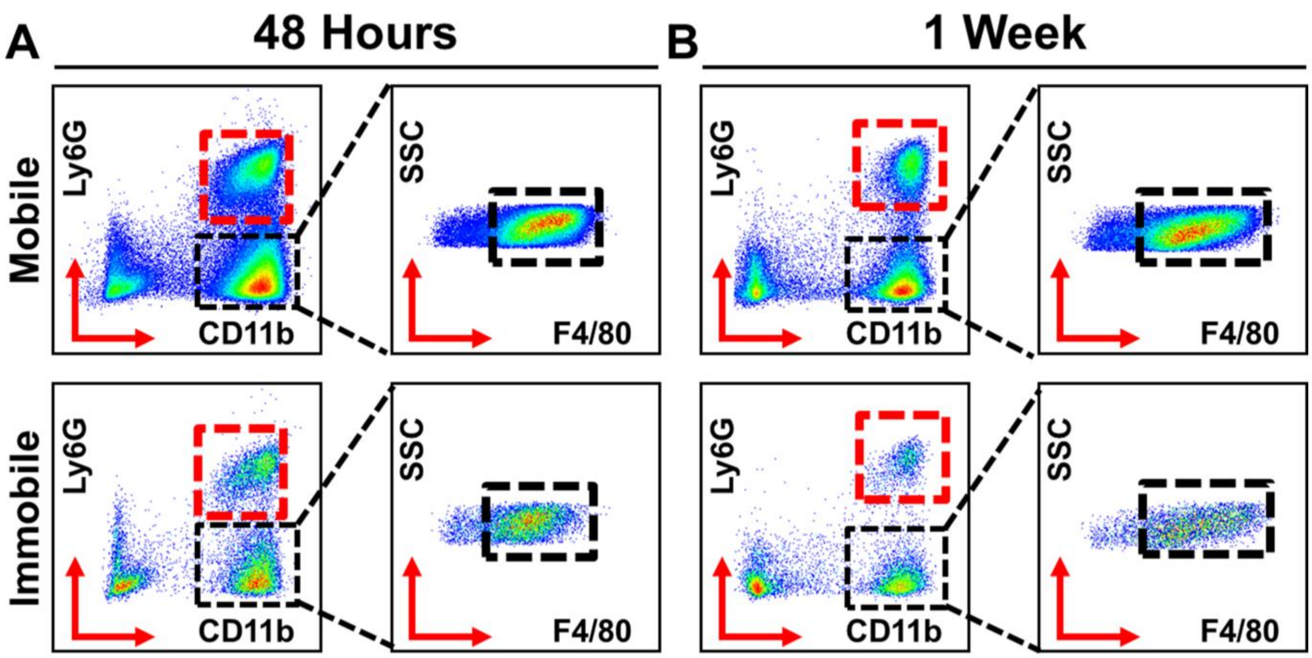

Supplemental Data Fig 3. (A) Flow cytometry gating strategy of mobile and immobile hindlimb samples 48 hours after injury (red dashed box indicates neutrophils; black dashed box indicates macrophages); (B) Flow cytometry gating strategy of mobile and immobile hindlimb samples 1 week after injury (red dashed box indicates neutrophils; black dashed box indicates macrophages).

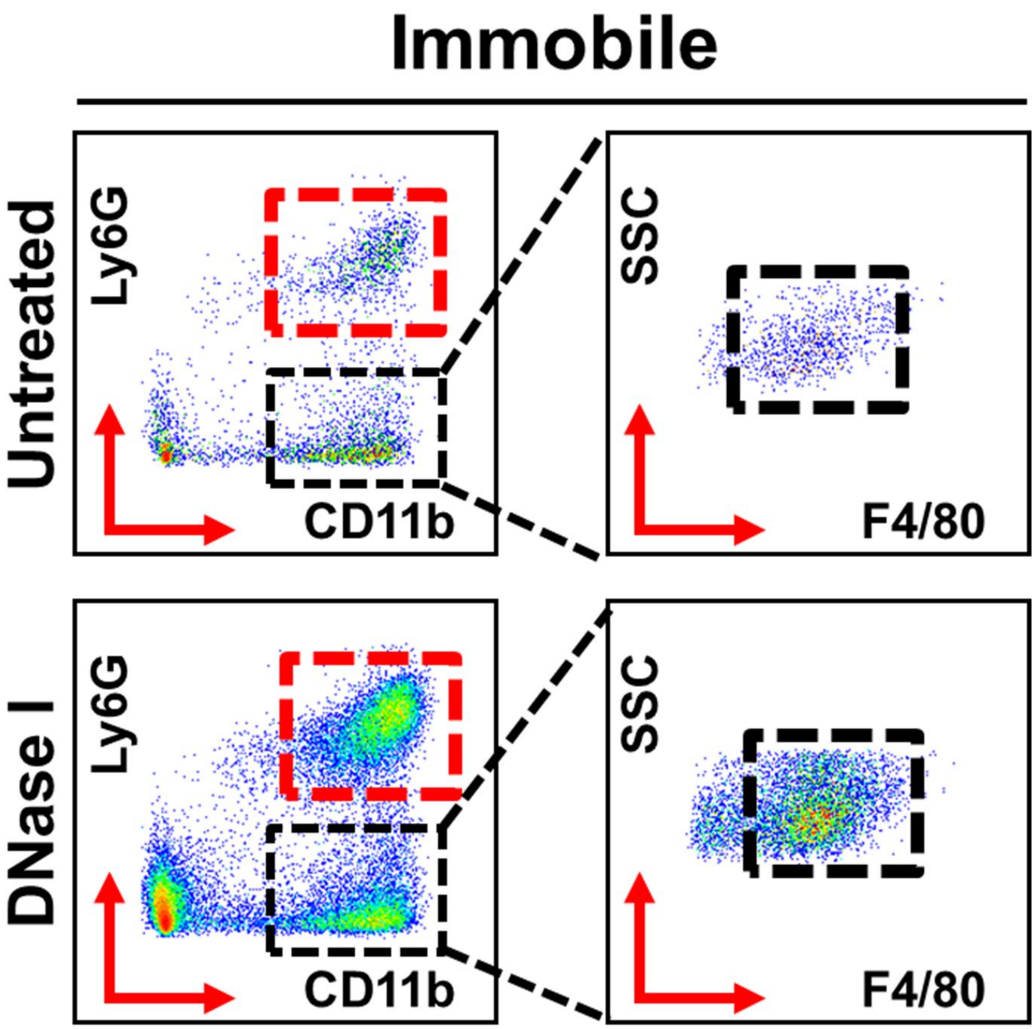

Supplemental Data Fig 4. Flow cytometry gating strategy of immobile hindlimb samples with or without DNase I treatment 48 hours after injury (red dashed box indicates neutrophils; black dashed box indicates macrophages).

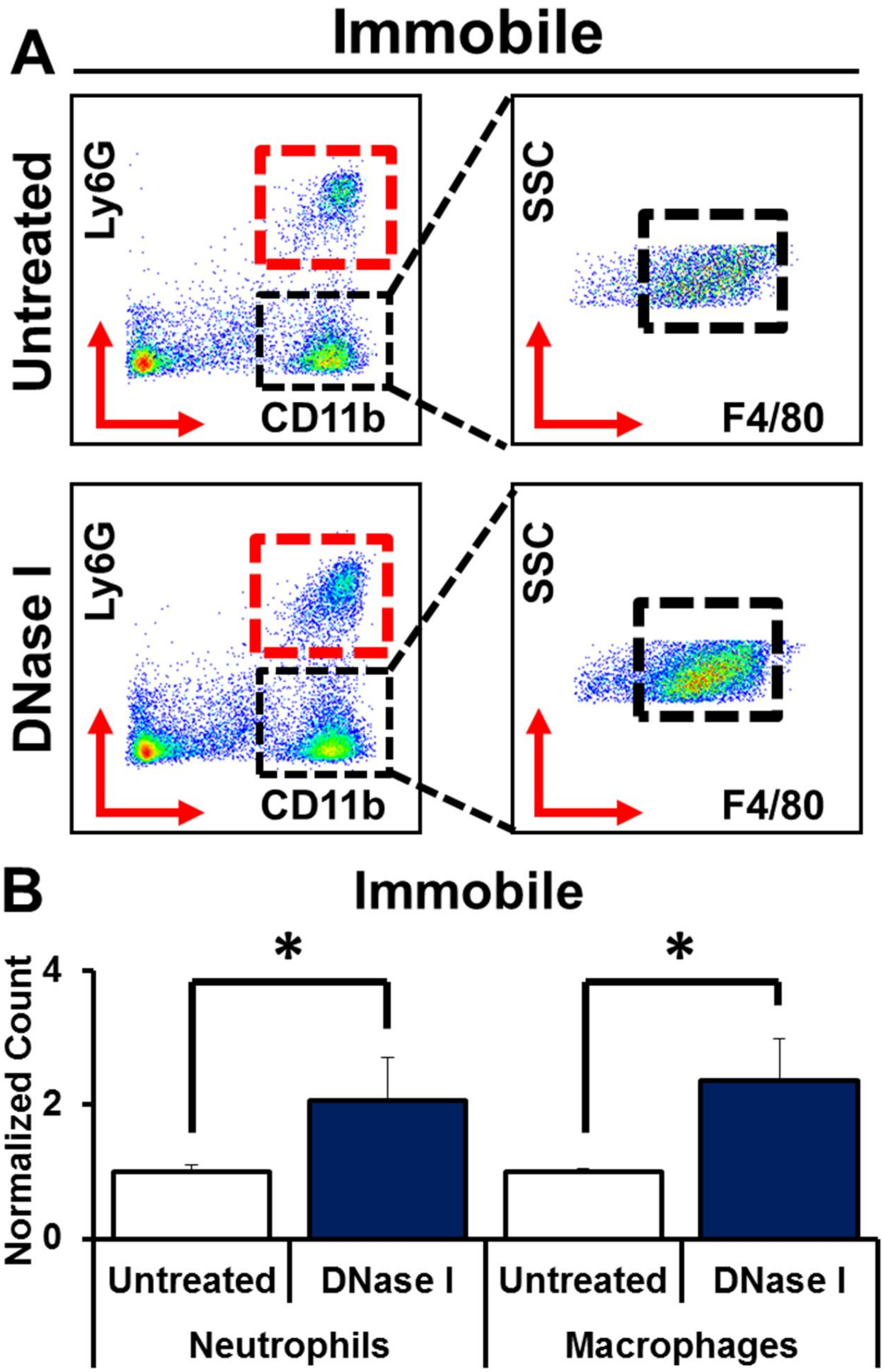

Supplemental Data Fig 5. (A) Flow cytometry gating strategy of immobile hindlimb samples with or without DNase I treatment 1 week after injury (red dashed box indicates neutrophils; black dashed box indicates macrophages); (B) DNase I treatment (n=3) significantly increases normalized neutrophil (1.0 v. 2.1, $p<0.05$) and macrophage (1.0 v. 2.4, $p<0.05$) counts in the immobile hindlimb 1 week after injury versus untreated controls (n=3).

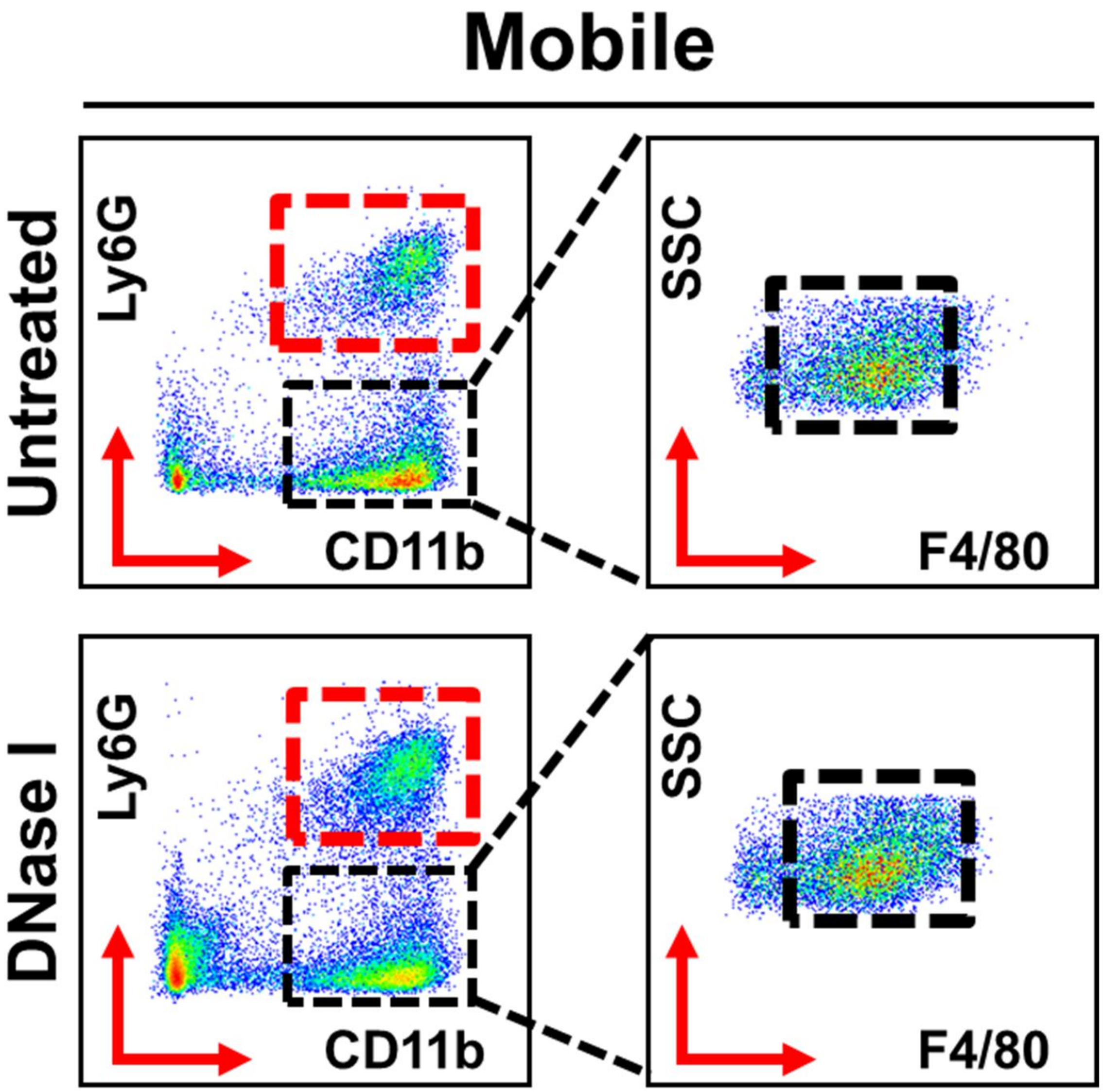

Supplemental Data Fig 6. Flow cytometry gating strategy of mobile hindlimb samples with or without DNase I treatment 48 hours after injury (red dashed box indicates neutrophils; black dashed box indicates macrophages).

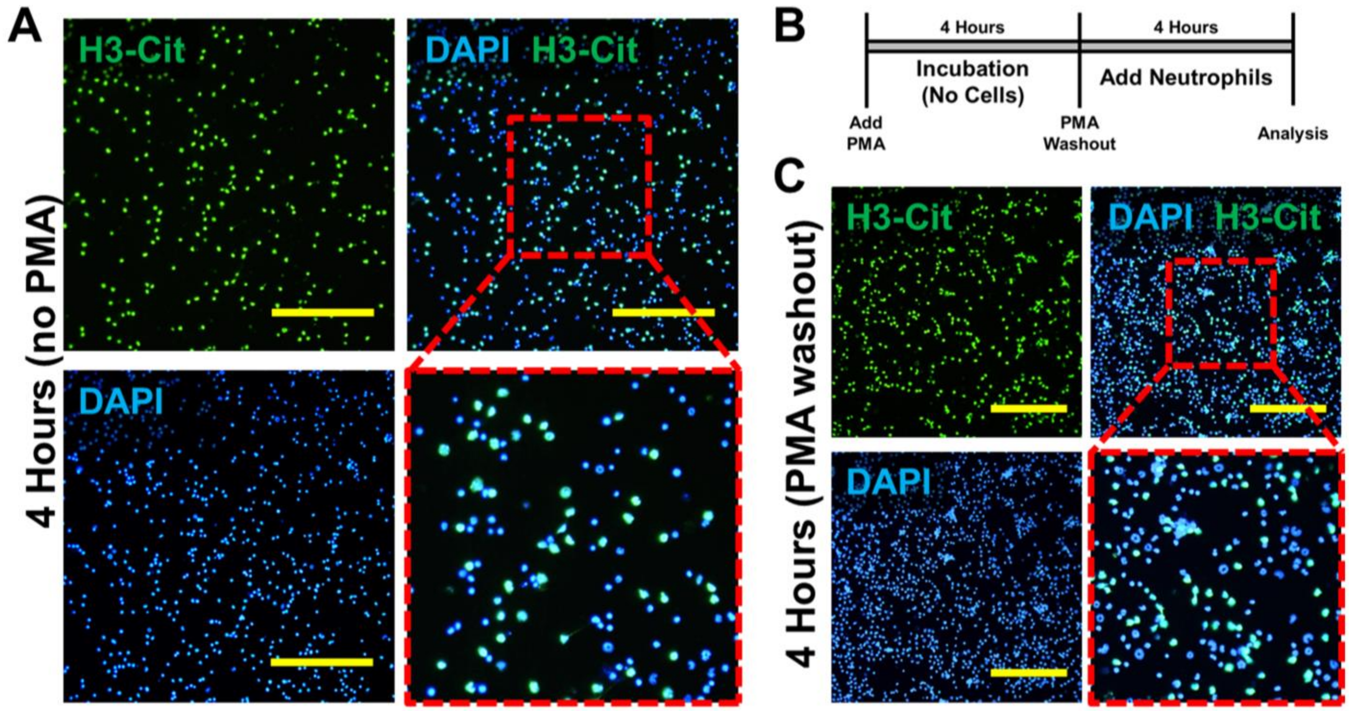

Supplemental Data Fig 7. (A) Control experiment showing absence of structures with NET morphology when 1° neutrophils in media are not exposed to PMA; (B) *In vitro* experimental design to verify that media change effectively removes residual PMA; (C) Control experiment showing absence of structures when PMA and media without 1° neutrophils are removed and 2° neutrophils are added. Scale bars are 200µm.

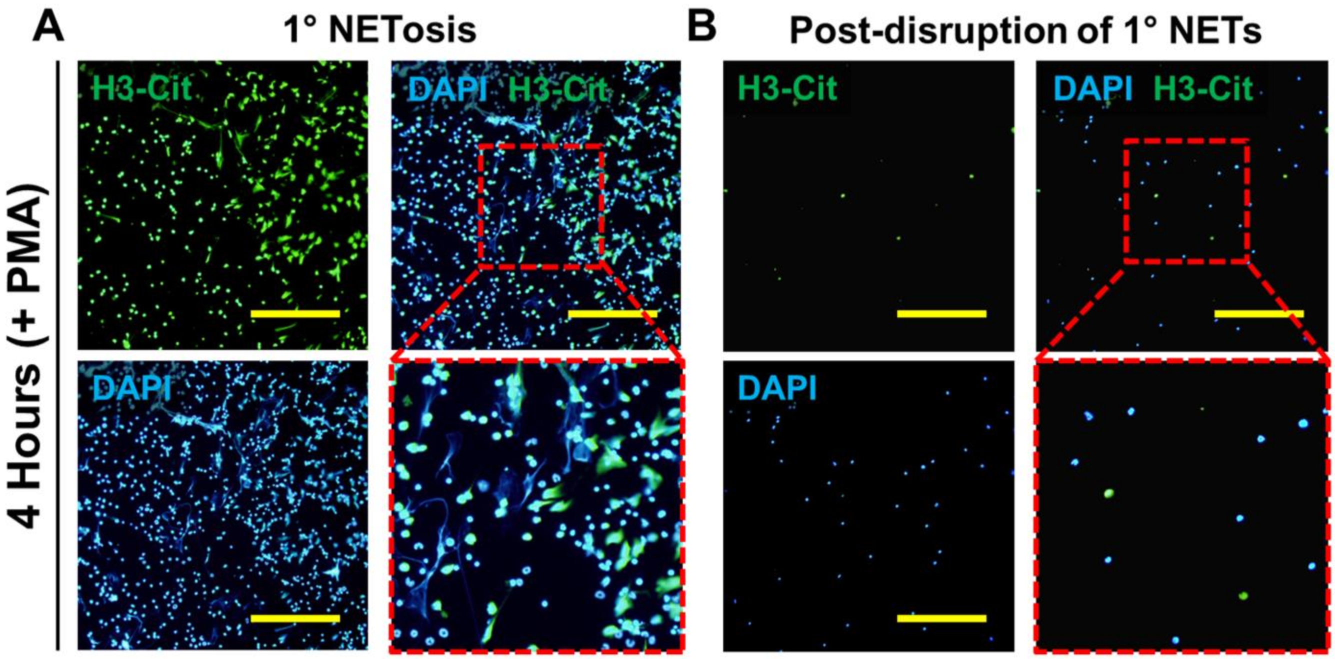

Supplemental Data Fig 8. (A) 1° neutrophils exposed to PMA undergo NETosis with visible NET structures; (B) Control experiment showing that gentle pipetting to disrupt 1° NETs eliminates visible NET structures. Scale bars are 200µm.

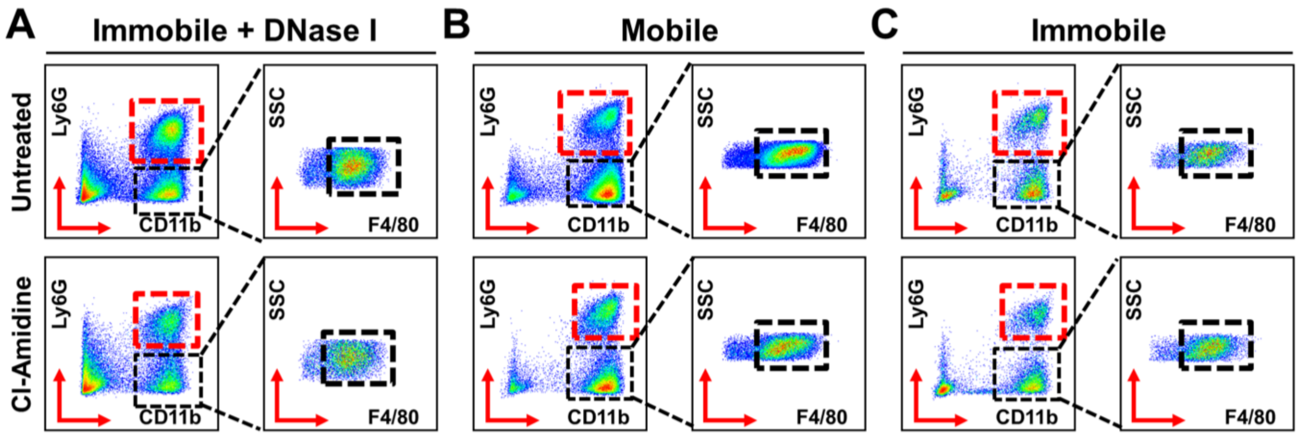

Supplemental Data Fig 9. (A) Flow cytometry gating strategy of immobile hindlimbs from mice 48 hours after injury, with immediate Cl-Amidine and DNase I treatment; (B) Flow cytometry gating strategy of mobile hindlimbs from mice 48 hours after injury, with immediate Cl-Amidine and DNase I treatment; (C) Flow cytometry gating strategy of immobile hindlimbs from mice 48 hours after injury, with immediate Cl-Amidine and DNase I treatment.

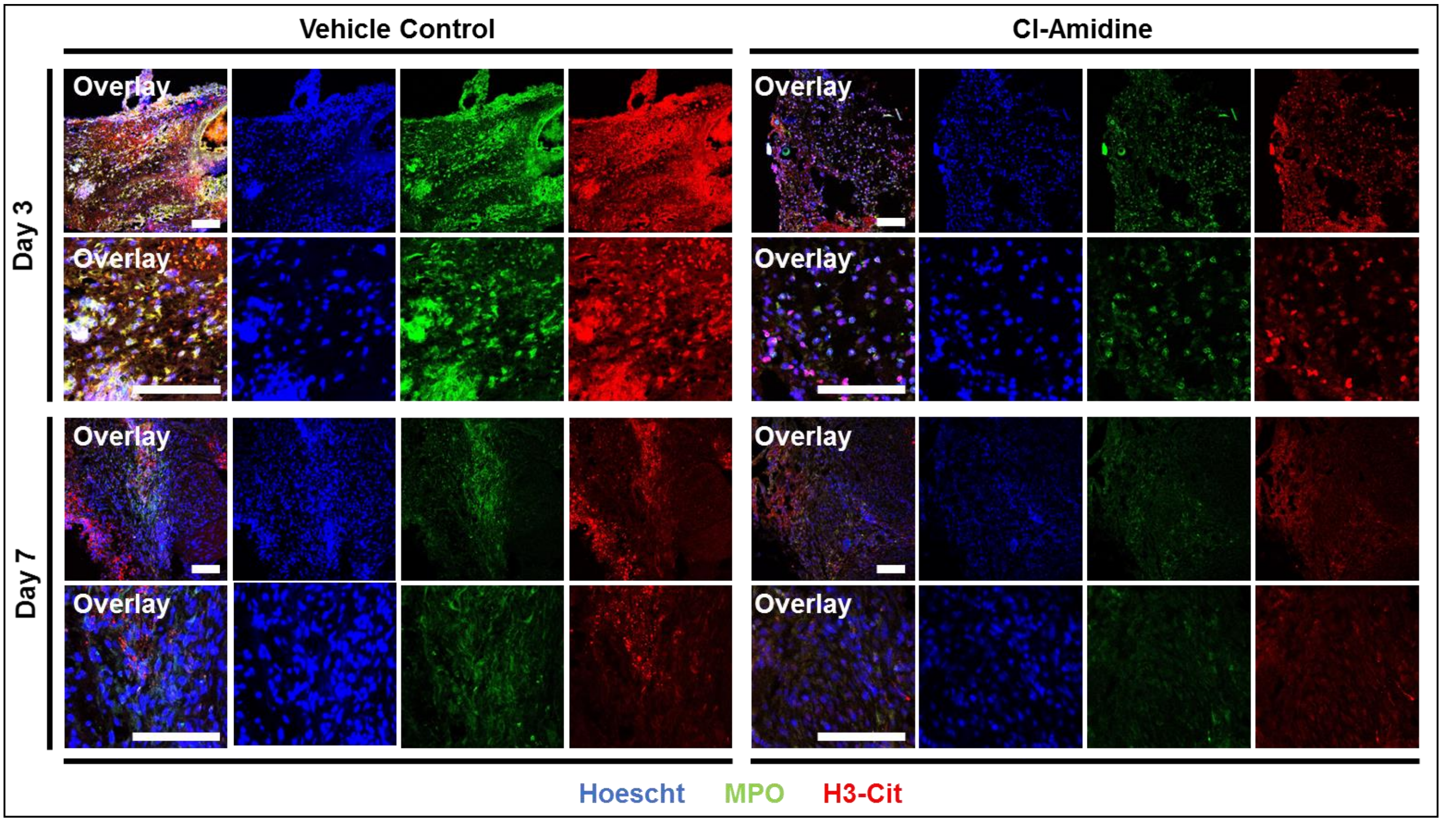

Supplemental Data Fig 10. PAD4 inhibition via CL-Amididine treatment attenuates formation of NETs at the site of injury. Representative micrographs of fluorescently immunolabeled injury site (HO anlagen) at day 3 and day 7 after inductive burn tenotomy. Intra- and extra-cellular overlap of citrullinated histones and myeloperoxidase represent NET formation (yellow in overlay) in Cl-Amidine and vehicle treated animals. n=3/group/timepoint. Scalebars indicate 100 µm.

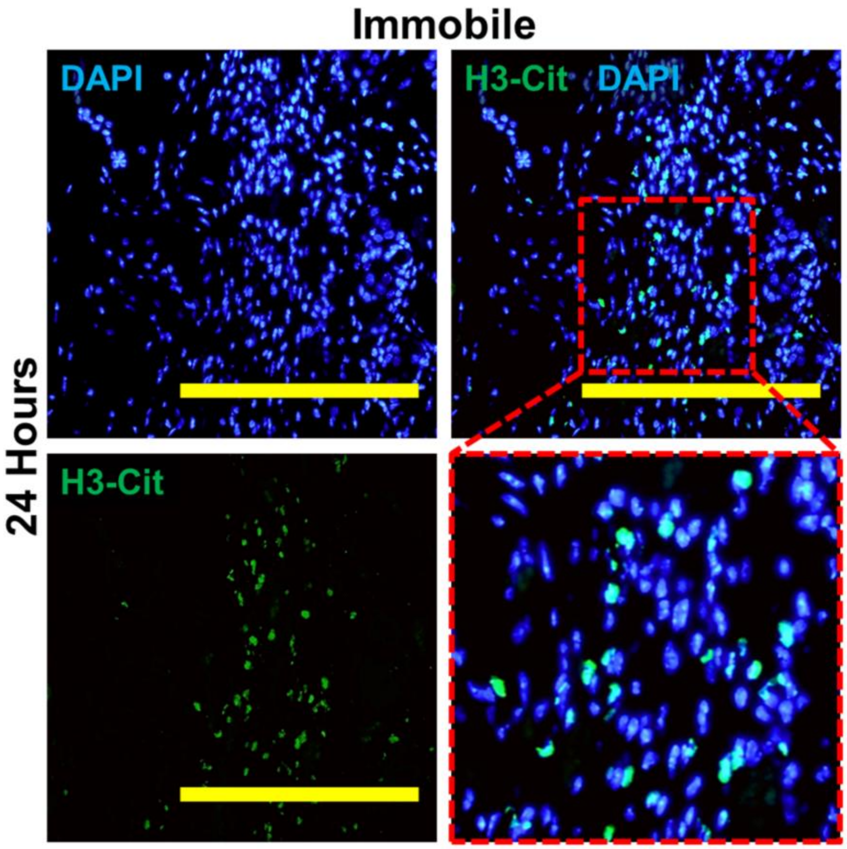

Supplemental Data Fig 11. Presence of H3-Cit+/DAPI+ NETs in histologic sections of the immobile hindlimb 24 hours after injury. Scale bars are 200µm.

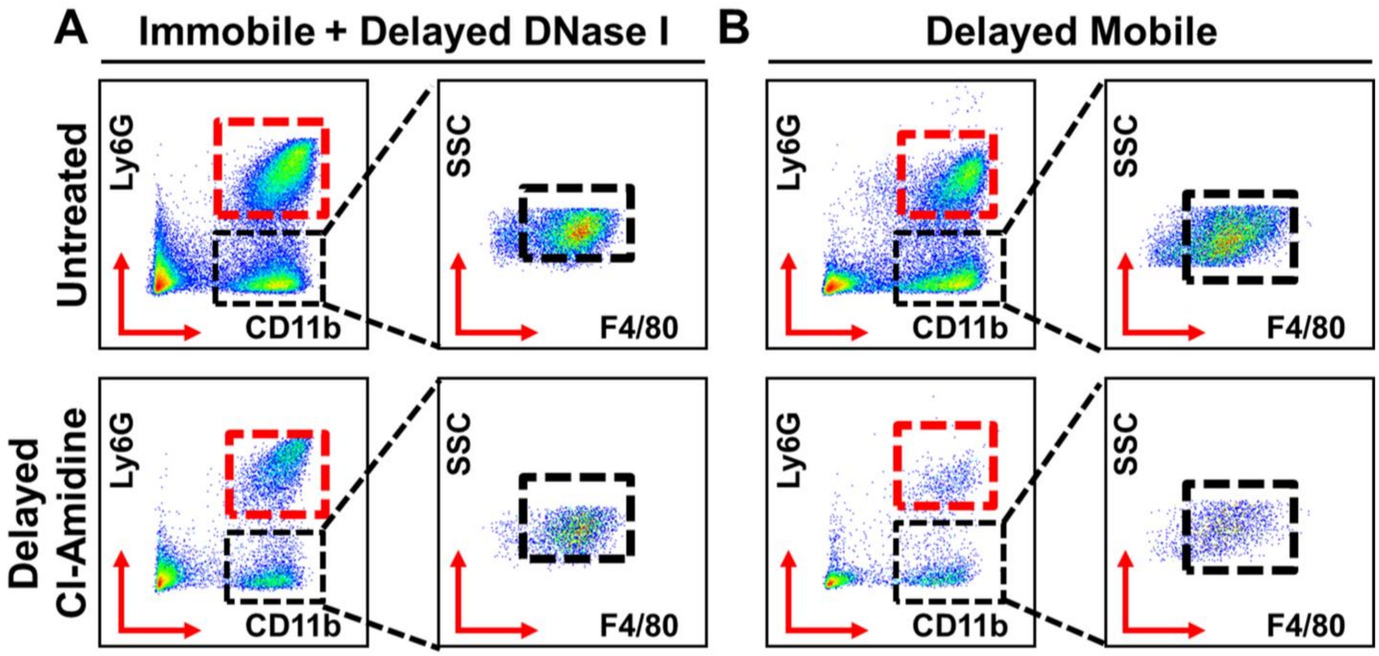

Supplemental Data Fig 12. (A) Flow cytometry gating strategy of immobile hindlimbs 48 hours after injury with delayed DNase I and Cl-Amidine treatment administered 24 hours after injury; (B) Flow cytometry gating strategy of hindlimbs immobilized for the first 24 hours after injury with subsequent removal of the immobilizer and Cl-Amidine treatment 24 hours after injury.

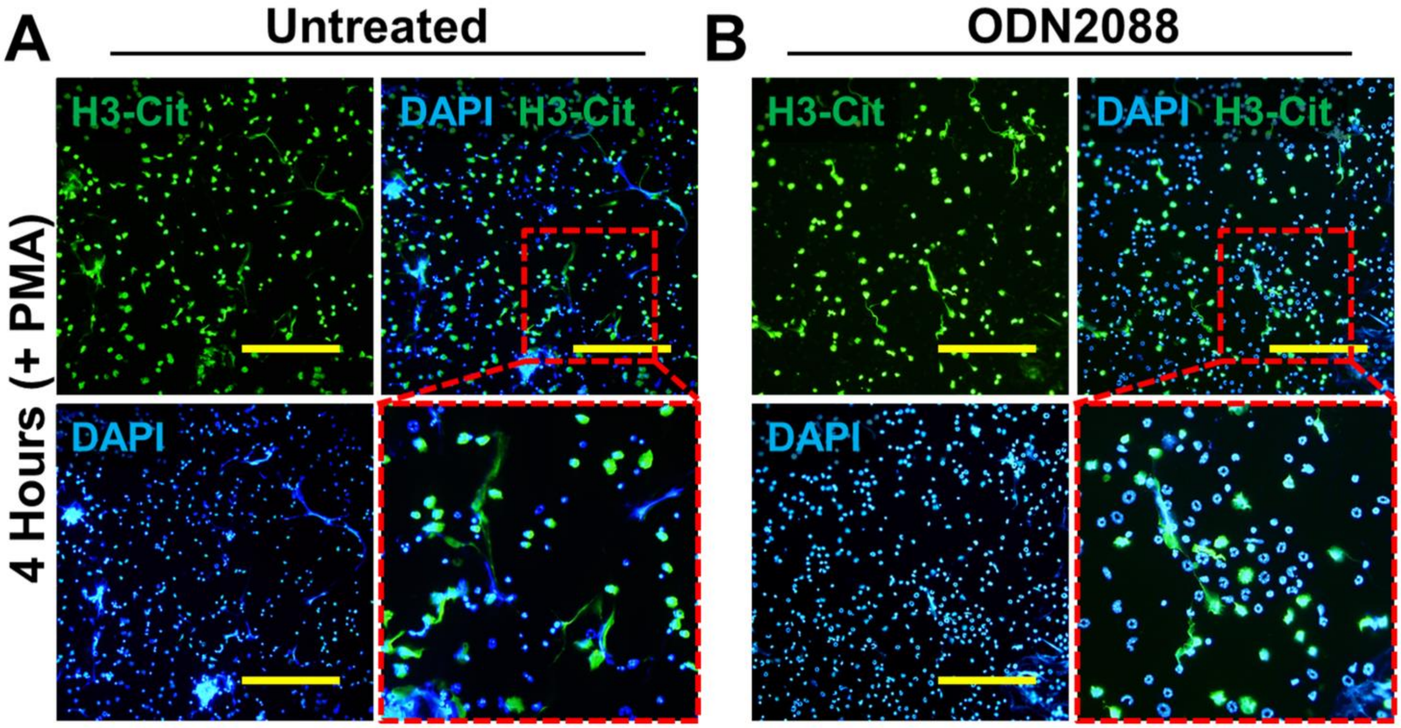

Supplemental Data Fig 13. (A) Neutrophils exposed to PMA for 4 hours form NETs; (B) ODN-2088 does not prevent neutrophils from undergoing PMA-induced NETosis. Scale bars are 200μm.

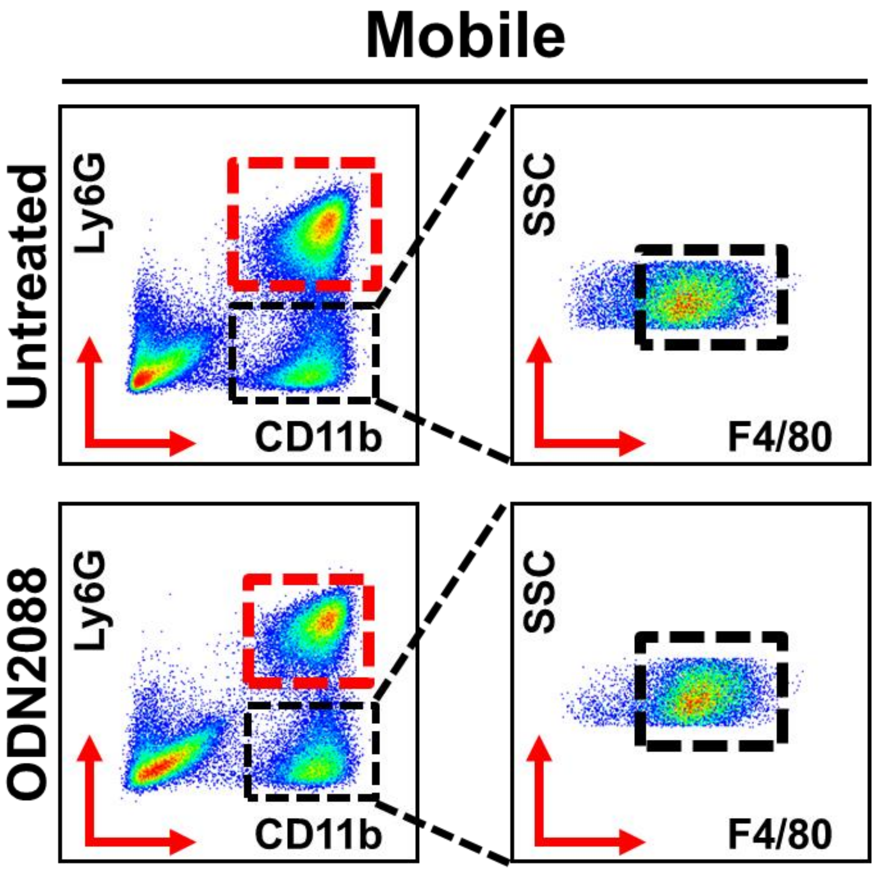

Supplemental Data Fig 14. (A) Flow cytometry gating strategy of mobile hindlimbs from ODN-2088-treated mice 48 hours after injury.

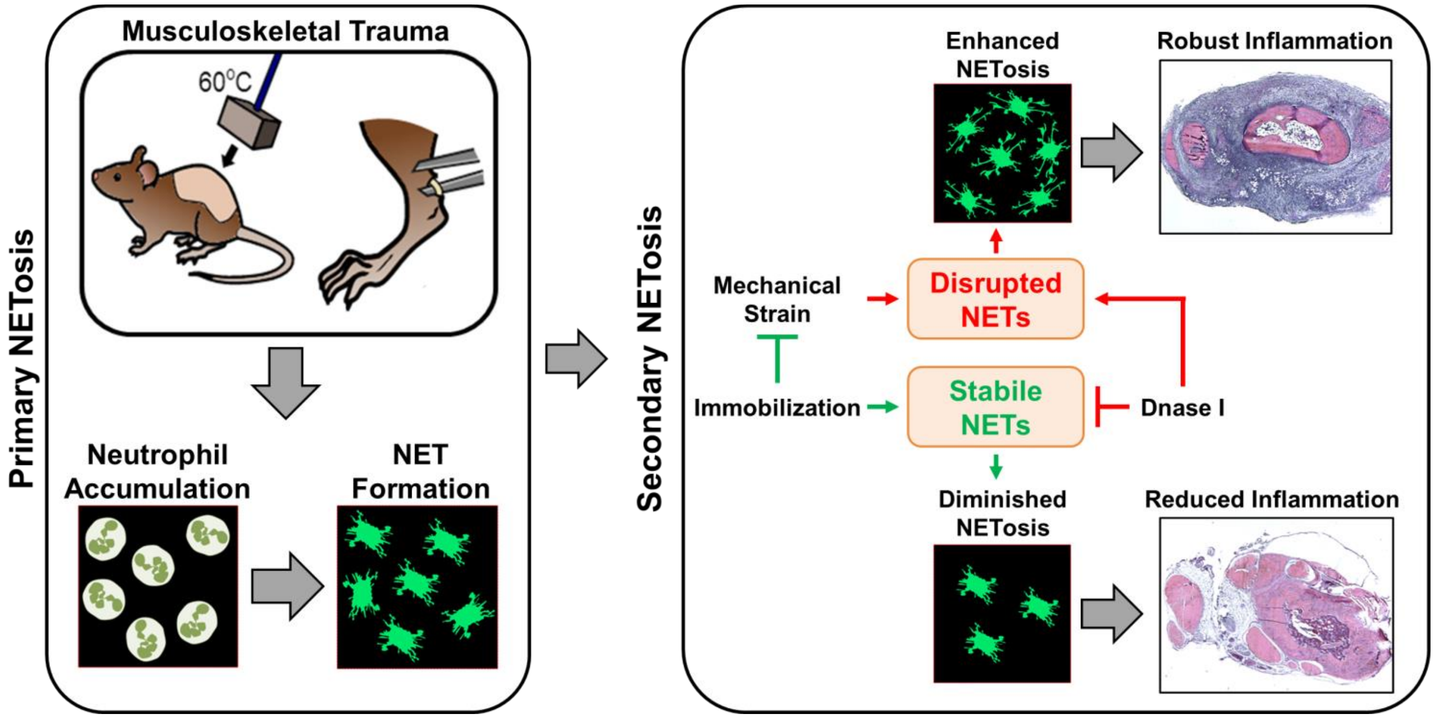

Supplemental Data Fig 15. Visual Abstract representing the generation of primary NETs after trauma and the differential progression to secondary NETosis induced by either disruption or stabilization of NETs.
